# Bilateral Acute Retinal Pigment Epitheliitis Diagnosed and Monitored Using Multimodal Imaging: A Case Report

**DOI:** 10.7759/cureus.109087

**Published:** 2026-05-18

**Authors:** Ayumi Kusano, Akira Watanabe, Kokoro Konuma, Tadashi Nakano

**Affiliations:** 1 Ophthalmology, The Jikei University School of Medicine, Tokyo, JPN

**Keywords:** acute retinal pigment epitheliitis, bilateral involvement, multimodal imaging, optical coherence tomography, white dot syndromes

## Abstract

We report a rare case of bilateral acute retinal pigment epitheliitis (ARPE) documented using multimodal imaging, with detailed sequential optical coherence tomography (OCT) findings. A woman in her 30s presented with acute bilateral visual deterioration without prodromal symptoms. Fundus examination revealed yellowish-white lesions localized to the fovea in both eyes. OCT demonstrated dome-shaped hyperreflective lesions with disruption of the ellipsoid zone (EZ) and interdigitation zone (IZ). OCT angiography, fluorescein angiography, and indocyanine green angiography showed no significant abnormalities. Based on these findings, bilateral ARPE was diagnosed. Given the bilateral visual impairment, topical betamethasone sodium phosphate was initiated to facilitate early recovery. Sequential OCT demonstrated gradual restoration of the outer retinal layers, with recovery of the external limiting membrane, followed by the IZ and EZ. Best-corrected visual acuity improved to 1.0 in both eyes within two months, and near-complete anatomical resolution was confirmed by three months. No recurrence was observed after treatment cessation. This case highlights the rare bilateral presentation of ARPE and demonstrates the value of OCT in monitoring layer-by-layer recovery of the outer retina, while emphasizing the importance of differentiating it from other white dot syndromes.

## Introduction

Acute retinal pigment epitheliitis (ARPE) was first described by Krill and Deutman in 1972 [1]. It is a rare, self-limiting inflammatory retinal disorder that typically affects healthy young to middle-aged individuals. ARPE is characterized by acute visual disturbances and central scotoma, often accompanied by characteristic funduscopic findings of fine pigment stippling surrounded by yellowish halos in the macular region [1,2]. ARPE most commonly presents unilaterally; however, bilateral involvement has been reported in approximately 9.8% of cases [2].

Optical coherence tomography (OCT) plays a key role in diagnosis, typically demonstrating hyperreflective abnormalities at the level of the photoreceptor outer segments and disruption of the ellipsoid zone (EZ) [2,3]. The condition usually resolves spontaneously within several weeks, with a favorable visual prognosis [3,4].

However, recent advances in multimodal imaging have raised questions regarding whether ARPE represents a distinct clinical entity or part of a broader spectrum of outer retinal disorders [2].

In this report, we describe a rare case of bilateral ARPE evaluated using multimodal imaging, including OCT, optical coherence tomography angiography (OCTA), fluorescein angiography (FA), and indocyanine green angiography (ICGA).

## Case presentation

A woman in her 30s presented with bilateral visual deterioration of one week’s duration. She reported no preceding flu-like symptoms and had no significant past medical history.

**Figure 1:**
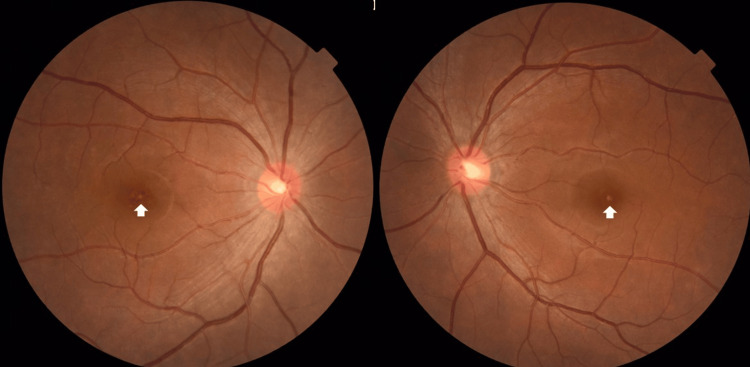
Color fundus photographs at the initial visit. Color fundus photographs of both eyes obtained at presentation show irregularly bordered, round, yellowish-white lesions localized to the fovea. White arrows indicate the lesions of interest.

At presentation, the best-corrected visual acuity (BCVA) was 0.5 in both eyes. Intraocular pressure was 15 mmHg in the right eye and 14 mmHg in the left eye. Intraocular pressure was measured using a non-contact tonometer. Slit-lamp examination revealed no anterior chamber inflammation. Fundus examination demonstrated irregularly bordered, round, yellowish-white lesions localized to the fovea in both eyes (Figure 1).

**Figure 2:**
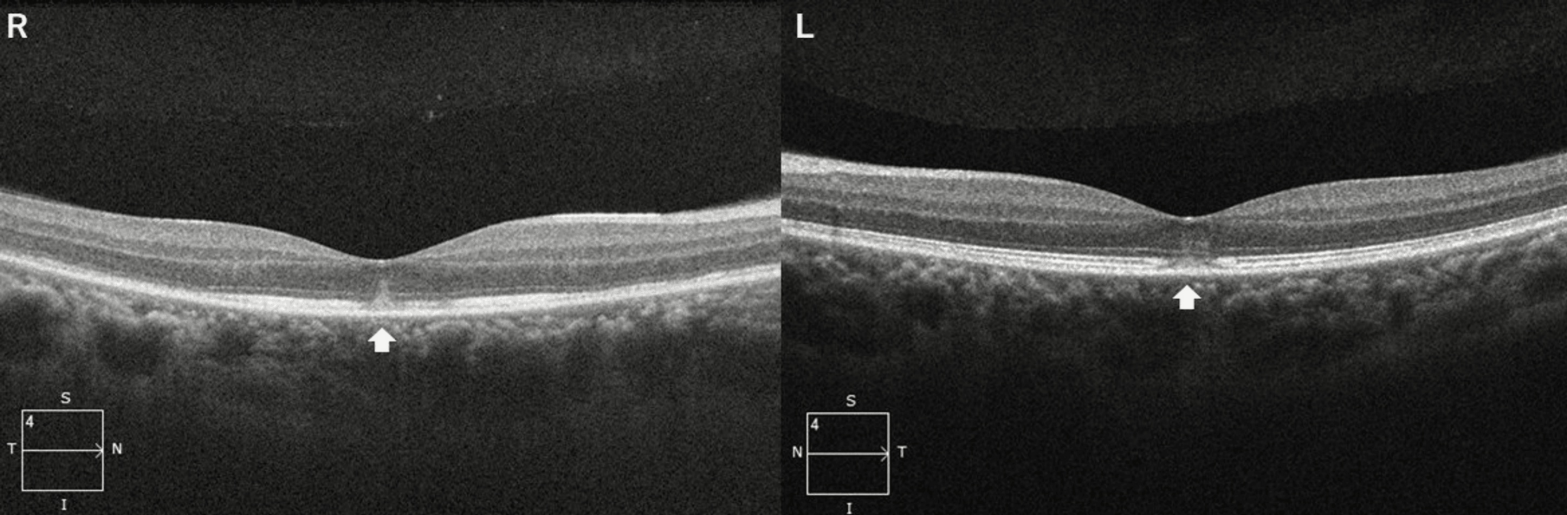
Optical coherence tomography findings at the initial visit. Horizontal optical coherence tomography (OCT) images of both eyes at presentation demonstrate dome-shaped hyperreflective lesions beneath the fovea, associated with disruption of the ellipsoid zone (EZ) and interdigitation zone (IZ). White arrows indicate the lesions of interest.

OCT revealed dome-shaped hyperreflective lesions beneath the fovea, with disruption of the EZ and interdigitation zone (IZ) in both eyes (Figure 2).

**Figure 3:**
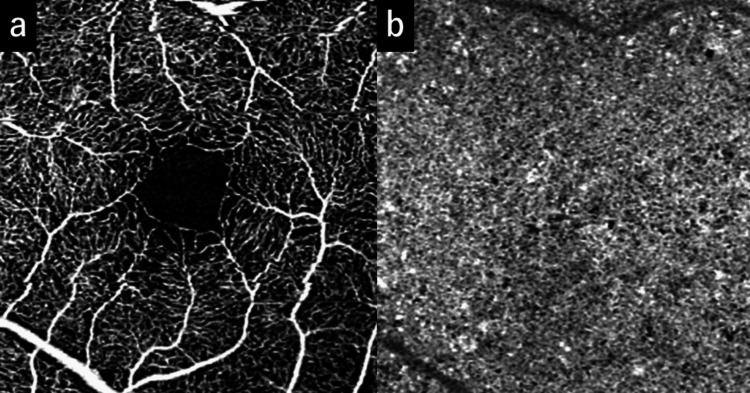
Optical coherence tomography angiography findings at the initial visit. Optical coherence tomography angiography images of the macular region show no significant abnormalities in the superficial vascular plexus (a) or choriocapillaris (b) in either eye.

OCTA showed no significant abnormalities in the macular region (Figure 3).

**Figure 4:**
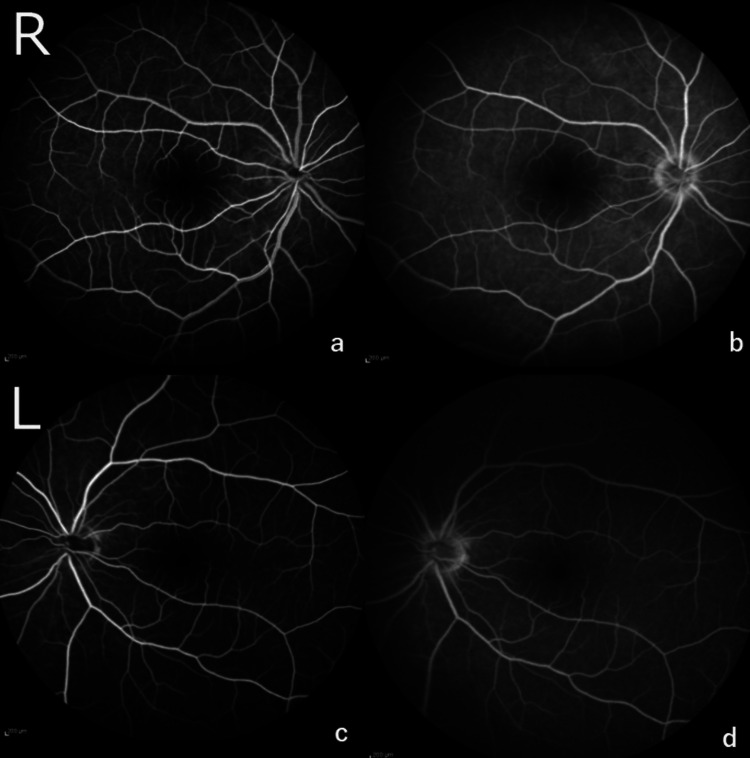
Fluorescein angiography findings at the initial visit: (a) early phase and (b) late phase of the right eye; (c) early phase and (d) late phase of the left eye. Fluorescein angiography demonstrates no remarkable leakage or choroidal abnormalities in either eye.

**Figure 5:**
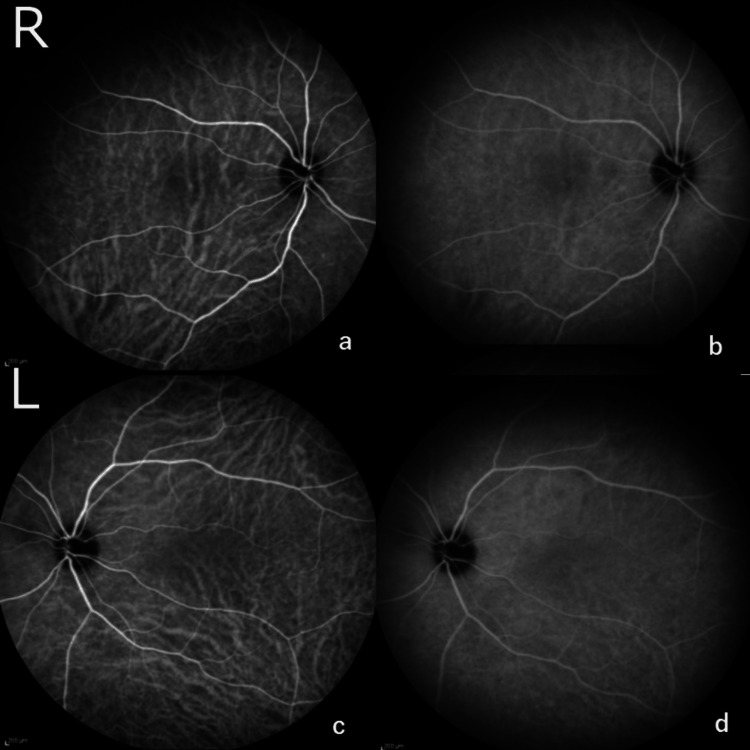
Indocyanine green angiography findings at the initial visit: (a) early phase and (b) late phase of the right eye; (c) early phase and (d) late phase of the left eye. Indocyanine green angiography demonstrates no remarkable leakage or choroidal abnormalities in either eye.

FA and ICGA also demonstrated no remarkable leakage or choroidal abnormalities. Blood examinations were unremarkable (Figures 4-5).

Based on these findings, a diagnosis of bilateral ARPE was made. Given the bilateral decrease in visual acuity and the expectation of early recovery, topical betamethasone sodium phosphate was initiated. Topical betamethasone sodium phosphate was administered to both eyes three times daily for two months. The treatment was discontinued without tapering after improvement in visual acuity, and the OCT findings were confirmed.

Clinical course

**Figure 6:**
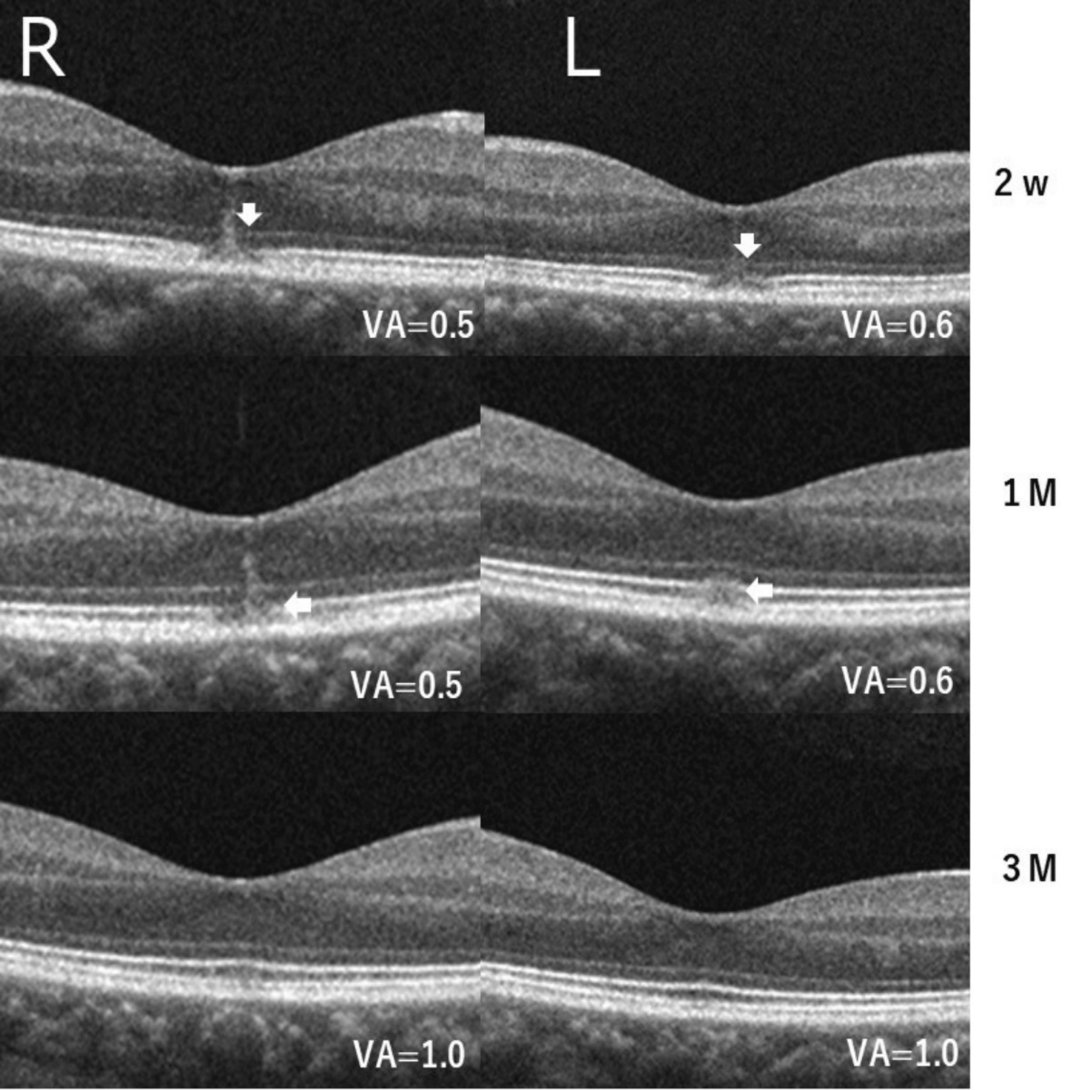
Sequential optical coherence tomography findings during follow-up. Serial optical coherence tomography (OCT) images show gradual restoration of the outer retinal layers in both eyes. Disruption of the external limiting membrane (ELM) improved first, followed by recovery of the interdigitation zone (IZ) and ellipsoid zone (EZ), with near-complete anatomical resolution by three months. White arrows indicate the areas of outer retinal disruption and subsequent recovery. VA, visual acuity

At the initial visit, OCT revealed that the disruptions extended to the EZ, IZ, and external limiting membrane (ELM) in both eyes. At this time, BCVA was 0.5 in both eyes. Two weeks later, disruption of the ELM gradually improved, and complete recovery of the ELM was observed at one month; however, BCVA remained 0.5 in the right eye and 0.6 in the left eye. At two months, the disruption of the IZ had resolved, while the slight disruption of the EZ persisted; nevertheless, BCVA had improved to 1.0 in both eyes. By three months, the disruption of the EZ had also completely resolved. Fundus and OCT findings showed near-complete resolution at three months. No recurrence was observed after cessation of treatment (Figure 6).

## Discussion

ARPE is a rare, typically unilateral, self-limiting disorder [1-4]; however, bilateral involvement and recurrence have been reported in a few cases [5,6]. Although a preceding viral illness or following vaccination has been suggested in some patients [7], no prodromal symptoms were identified in the present case. This case was characterized by bilateral involvement and detailed documentation of sequential structural recovery using OCT.

A key finding in this case was the layer-by-layer recovery of the outer retina. OCT initially demonstrated disruption of the ELM, IZ, and EZ, followed by gradual restoration of the ELM, IZ, and EZ. This sequential recovery pattern may reflect the process of photoreceptor structural repair and is closely associated with improvements in visual acuity. To our knowledge, detailed temporal documentation of layer-specific recovery in ARPE remains limited.

Although ARPE is generally considered a self-limiting condition with complete recovery of visual acuity and OCT findings, recent studies using adaptive optics imaging have suggested that parafoveal cone density may remain reduced even after apparent structural recovery [8]. This indicates that subclinical photoreceptor damage may persist despite normalization of conventional imaging findings.

These findings highlight the value of OCT not only for diagnosis but also for monitoring disease progression and recovery.

Multimodal imaging plays an essential role in establishing the diagnosis and excluding other conditions. The absence of leakage on FA and no significant abnormalities on OCTA supported the diagnosis of ARPE. Unlike other white dot syndromes, such as multiple evanescent white dot syndrome (MEWDS), acute posterior multifocal placoid pigment epitheliopathy, and punctate inner choroidopathy, ARPE is characterized by foveal localization, the absence of angiographic leakage, and isolated outer retinal disruption on OCT.

Recent literature has raised important questions regarding whether ARPE represents a distinct clinical entity [2,9]. A critical review by Al-Nofal and Charbel Issa [9] suggested that many cases previously diagnosed as ARPE could be reclassified into other well-defined conditions, including MEWDS, central serous chorioretinopathy (CSC)/pachychoroid spectrum disease, and acute macular neuroretinopathy (AMN). Based on these observations, ARPE may represent a diagnostic construct originating from the pre-OCT era rather than a true independent disease entity.

In contrast, other authors have argued that ARPE should not be dismissed as a diagnostic myth [10]. Although acknowledging the heterogeneity and lack of consensus in its definition, they emphasize that certain clinical and imaging features cannot be fully explained by existing disease entities. In particular, subfoveal outer retinal hyperreflectivity and retinal pigment epithelium (RPE)-level abnormalities observed in presumed ARPE cases are not entirely consistent with the phenotypes of AMN, MEWDS, or CSC, suggesting that ARPE may represent a distinct, self-limiting disorder affecting the outer retina and RPE interface.

More recently, ARPE has been proposed to represent a spectrum of disease with variable clinical course and prognosis rather than a single uniform entity. Ahmed-Balestra et al. suggested a classification based on age, laterality, and multimodal imaging findings, dividing ARPE into milder unilateral forms with favorable recovery and more severe bilateral forms, particularly in younger patients, that may be associated with persistent structural damage and incomplete visual recovery [11].

In this context, careful multimodal imaging is essential for accurate diagnosis and differential diagnosis. Solar retinopathy was also considered in the differential diagnosis because bilateral foveal lesions with outer retinal hyperreflectivity and EZ disruption on OCT may resemble the findings observed in the present case. However, the absence of a history of solar exposure and the rapid restoration of the EZ within three months were considered atypical for solar retinopathy.

In the present case, the absence of widespread white dots or hyperfluorescent lesions on fluorescein angiography made MEWDS unlikely. Similarly, no evidence of serous retinal detachment, choroidal thickening, or choroidal hyperpermeability was observed, arguing against CSC or pachychoroid spectrum disease. Furthermore, the lesion was localized to the fovea, which is atypical for AMN, where lesions are usually parafoveal.

A notable feature of this case was the bilateral involvement and the clearly documented sequential recovery of the outer retinal layers on OCT. Disruption initially involved the external limiting membrane (ELM), IZ, and EZ, followed by gradual restoration in a layer-by-layer manner. This temporal sequence may reflect the process of photoreceptor structural repair and correlated well with visual recovery. While such findings are consistent with previously reported ARPE features, they may also overlap with other transient outer retinal disorders.

Interestingly, although bilateral involvement has been associated with more severe phenotypes and poorer visual outcomes in some reports, the present case demonstrated favorable visual recovery despite bilateral disease. This finding may suggest the existence of intermediate or milder bilateral phenotypes within the proposed ARPE spectrum.

Therefore, rather than definitively categorizing this case as a distinct entity, it may be more appropriate to interpret it within the spectrum of self-limiting disorders affecting the outer retina and RPE interface. At the same time, the presence of cases that cannot be fully explained by currently recognized entities supports the possibility that ARPE represents a unique clinical phenotype within this spectrum.

Taken together, this case highlights both the diagnostic challenges and the evolving conceptual understanding of ARPE in the era of multimodal imaging. Further accumulation of well-characterized cases using comprehensive imaging modalities will be essential to refine the diagnostic criteria and clarify the clinical boundaries of ARPE.

Although ARPE is generally considered self-limiting, topical corticosteroid therapy was initiated in this case because of bilateral visual impairment and the expectation of facilitating early functional recovery. Because inflammatory involvement of the outer retina and RPE interface has been suggested in presumed ARPE, topical corticosteroid therapy was considered a low-invasive therapeutic option. However, given the natural course of ARPE, the extent to which corticosteroid therapy contributed to the observed improvement remains uncertain and may have been limited [12].

Previous reports have suggested that cases with an initial BCVA of less than 0.3, and those with OCT findings demonstrating involvement extending to the ELM or outer nuclear layer (ONL), may be associated with incomplete visual recovery even after prolonged follow-up [4]. In the present case, although the outer retinal layers, including the ELM, were involved at presentation, visual acuity showed favorable recovery, suggesting that structural restoration may precede or parallel functional improvement in some cases.

The extent of outer retinal involvement in ARPE has been reported to vary, with consistent involvement of the EZ and IZ and less frequent extension to the ELM or ONL [13]. The present case is consistent with these observations and demonstrates a clear temporal sequence of structural recovery. This case highlights the utility of OCT in elucidating the dynamic process of outer retinal repair and contributes to a more refined understanding of the structural-functional relationship in ARPE.

## Conclusions

This case demonstrates that presumed ARPE can present bilaterally and provides detailed documentation of the sequential, layer-by-layer recovery of the outer retina on OCT. These findings offer insight into the process of photoreceptor structural restoration and highlight the value of OCT in monitoring disease progression and recovery. At the same time, in light of the ongoing debate regarding the nature of ARPE, this case underscores the importance of careful differential diagnosis using multimodal imaging. Rather than definitively establishing ARPE as a distinct disease entity, the present findings may be more appropriately interpreted within the spectrum of self-limiting disorders affecting the outer retina and RPE interface.

Further accumulation of well-characterized cases using comprehensive imaging modalities will be essential to refine the diagnostic criteria and clarify the clinical boundaries of ARPE.
